# Supplementary material for: Topologically crafted spatiotemporal vortices in acoustics
Source: Nat Commun. 2023 Oct 6;14:6238. doi: 10.1038/s41467-023-41776-8 (PMC10558554; doi:10.1038/s41467-023-41776-8)
Supplement: Supplementary file 1 — Supplementary Information [file 41467_2023_41776_MOESM1_ESM.pdf]

Supplementary Information

Topologically crafted spatiotemporal vortices in acoustics

Hongliang Zhang[1#], Yeyang Sun[1#], Junyi Huang[1#], Bingjun Wu[1],
Zhaoju Yang[1*], Konstantin Y. Bliokh[2,3,4], and Zhichao Ruan[1,5**]

[1]*School of Physics, Zhejiang Province Key Laboratory of Quantum Technology and Device, and State Key Laboratory for Extreme Photonics and Instrumentation, Zhejiang University, Hangzhou 310027, China*
[2]*Theoretical Quantum Physics Laboratory, RIKEN Cluster for Pioneering Research, Wako-shi, Saitama 351-0198, Japan*
[3]*Centre of Excellence ENSEMBLE3 Sp. z o.o., 01-919 Warsaw, Poland*
[4]*Donostia International Physics Center (DIPC), Donostia-San Sebastián 20018, Spain*
[5]*College of Optical Science and Engineering, Zhejiang University, Hangzhou 310027, China*

[#] These authors equal contributions to this work
* zhaojuyang@zju.edu.cn
** zhichao@zju.edu.cn

S1. The details of geometry parameters of acoustic spatiotemporal vortex pulse generator

We consider a mirror-asymmetric spatiotemporal vortex pulse (STVP) on the z-axis. Assume that there exists a STVP with the winding number of l generated by the meta-grating. Due to the existence of mirror symmetry about the axis $x = 0$, we can easily obtain that there must exist a STVP with the winding number of $-l$ that is generated together with the STVP with the winding number of l. Therefore, according to the uniqueness theorem of wave equations, we can say that only the wave solution with the winding number of $l = 0$ is satisfied. As a result, without loss of generality, we suppose that the necessary condition of generating STVPs with the nonzero winding number is mirror symmetry breaking, which provides a simultaneous control for sound in both spatial and temporal domains.

Block	Length(mm)	Width(mm)	A_i
B1	22.500	12.967	1.170
B2	23.330	10.290	9.500
B3	8.660	2.990	2.170
B4	16.580	3.320	-1.790

Table. S1 Structure parameters and shifting ratios A_i for four rectangular blocks in Fig. S1. The block shift toward right (left) when A_i is positive (negative).

The structure of the STVP generator consists of a fixed symmetrical air unit and four air blocks which are deformed to break mirror symmetry. The fixed part is marked in yellow in Fig. S1, and the geometric parameters are $d_1 = 5.670mm$, $d_2 = 10.000mm$, $d_3 = 7.670mm$, $h_1 = 12.997mm$, $h_3 = 16.663mm$, and $h_4 = 3.330mm$. For $\eta = 0$, the four blocks are spatially mirror-symmetrical with respect to the fixed part. When the four blocks are shifted to the left or right, the mirror symmetry of the structure is gradually broken. The offsets of the four blocks relative to the axis of symmetry are defined as: $\delta x_i = A_i \eta$, where A_i is the shifting ratio different for each block and η is a synthetic parameter characterizing the degree of asymmetry. The specific values of parameters for the four blocks and their corresponding shifting ratios are shown in Table. S1. This was not "derived" from any equations. The specific geometry and parameters of the metasurface are determined only by calculating the transmission spectrum function numerically and just following the symmetry breaking principle.

S2. The detailed derivation about STVP generation

In order to specifically depict the transformation between the incident and transmitted pulses while propagating through the STVP generator, we decompose the incident (transmitted) pulse envelope into a series of plane waves by Fourier transform $s_{in(out)}(x,t) = \iint \tilde{s}_{in(out)}(K_x, \Omega)\, exp(iK_x x - i\Omega t)\, dK_x d\Omega$, where $K_x = k_x - k_x^0$ is the wavevector component shifted along x direction, and $\Omega = \omega - \omega_0$ is the sideband angular frequency from the center one ω_0. In the polar coordinate,

the expression is converted to $s_{in(out)}(r,\theta) = \int_0^{\infty}\int_0^{2\pi} \tilde{s}_{in(out)}(\rho,\chi)\, exp[i\rho r\, cos(\chi+\theta)]\, \rho d\rho d\chi$ and where $r=\sqrt{x^2+v^2t^2}$, $\theta = tan^{-1}(vt/x)$, $\rho = \sqrt{{K_x}^2 + \Omega^2/v^2}$, $\chi = tan^{-1}(\Omega/vK_x)$, and v is the group velocity of acoustic wave. According to the restriction of the winding number around the vortex, within the small enough region, the amplitude of transmission spectrum function T for the STVP generator can be written as $f\,(\rho)$, and without loss of general T can be expressed as

$$T(\rho,\chi) = f\,(\rho)(Ae^{il\chi} + Be^{-il\chi}),$$

where l is the winding number of the vortex, A and B are the two constant parameters, respectively. We note that when the phase singularity is anisotropic, both A and B are nonzero. The overall topological charge is l if $|A|$ is larger than $|B|$, and $-l$ otherwise. Suppose that an incident pulse with zero topological charge $\tilde{s}_{\mathrm{in}}(\rho)$ impinges on the meta-gratings, the transmitted pulse envelope can be expressed as:

$$\begin{aligned} s_{out}(r,\theta) &= \iint \tilde{s}_{in}(\rho) f(\rho)\left(Ae^{il\chi} + Be^{-il\chi}\right)\cdot e^{i(K_x x-\Omega t)} dK_x d\Omega \\ &= \int_0^{\infty} \tilde{s}_{in}(\rho) f(\rho)\rho \int_0^{2\pi} Ae^{il\chi} e^{i(\rho\cos\chi\cdot r\cos\theta-\rho\sin\chi\cdot r\sin\theta)} + Be^{-il\chi} e^{i(\rho\cos\chi\cdot r\cos\theta-\rho\sin\chi\cdot r\sin\theta)} d\chi d\rho \\ &= \int_0^{\infty} \tilde{s}_{in}(\rho) f(\rho)\rho \left[Ae^{-il\theta}\int_0^{2\pi} e^{i[-l(-\chi-\theta)+\rho r\cos(\chi+\theta)]} d\chi + Be^{il\theta}\int_0^{2\pi} e^{i[-l(\chi+\theta)+\rho r\cos(\chi+\theta)]} d\chi \right] d\rho. \end{aligned}$$

Taking $\eta = -\chi - \theta + \frac{\pi}{2}$ and $\tau = \chi + \theta + \frac{\pi}{2}$, then $d\eta = -\, d\chi$ and $d\tau = d\chi$, by periodicity, the integral of $d\chi$ becomes:

$$\begin{aligned} & Ae^{-il\theta}\int_0^{2\pi} e^{i[-l(-\chi-\theta)+\rho r\cos(\chi+\theta)]} d\chi + Be^{il\theta}\int_0^{2\pi} e^{i[-l(\chi+\theta)+\rho r\cos(\chi+\theta)]} d\chi \\ &= -Ae^{-il\theta}\int_{-\theta+\frac{\pi}{2}}^{-2\pi-\theta+\frac{\pi}{2}} e^{i[-l\left(\eta-\frac{\pi}{2}\right)+\rho r\cos(\frac{\pi}{2}-\eta)]} d\eta + Be^{il\theta}\int_{\theta+\frac{\pi}{2}}^{2\pi+\theta+\frac{\pi}{2}} e^{i[-l\left(\tau-\frac{\pi}{2}\right)+\rho r\cos(\tau-\frac{\pi}{2})]} d\tau \\ &= -i^l Ae^{-il\theta}\int_0^{-2\pi} e^{i[-l\eta+\rho r\sin(\eta)]} d\eta + i^l Be^{il\theta}\int_0^{2\pi} e^{i[-l\tau+\rho r\sin(\tau)]} d\tau n \\ &= i^l Ae^{-il\theta}\int_0^{2\pi} e^{i[-l\eta+\rho r\sin(\eta)]} d\eta + i^l Be^{il\theta}\int_0^{2\pi} e^{i[-l\tau+\rho r\sin(\tau)]} d\tau \\ &= 2\pi i^l (Ae^{-il\theta} + Be^{il\theta}) J_l(\rho r) \end{aligned}$$

Where $J_l(\rho r) = \frac{1}{2\pi}\int_0^{2\pi} e^{i[-l\gamma+\rho r\, sin(\gamma)]} d\gamma$ is the *l-th* order Bessel function of the first kind. Therefore, the transmitted pulse envelope can be expressed as $s_{out}(r,\theta) = 2\pi i^l\left(Ae^{-il\theta} + Be^{il\theta}\right)\int_0^{\infty} \tilde{s}_{in}(\rho) f(\rho) J_l(\rho r)\rho d\rho$. Therefore, the transmitted wave packet can be determined to be

a STVP with a winding number of $-l$, which is opposite to that of the vortex in the $k_x - \omega$ domain.

S3. Transmission spectrum function of the structure with synthetic parameter $\eta = 1.0$

We measure the transmission spectrum function T of the unperturbed meta-grating with synthetic parameter $\eta = 1.0$ in the experiment and Fig. S6(a, b) show the amplitude and phase distribution of the transmission spectrum function with respect to $K_x = k_x - k_x^0$ and $\Omega = \omega - \omega_0$, respectively. The phase distribution of T exhibits that the vortex in the $k_x - \omega$ domain still survives, which leads to a zero amplitude at $\omega_0/2\pi = 8.02 \times 10^3 Hz$ and $k_{0x} = 0.01k_0$. Furthermore, the blue and orange circles in Fig. S6(c, d) correspond to the amplitudes and phases of the spectrum function along $K_x/k_0 = 0$ and $\Omega/\omega_0 = 0$ respectively.

We also measure the transmission spectrum function T of the perturbed spatiotemporal differentiator which has a block in each unit cell and the randomly placed fifteen particles (Fig. 3c). Similarly, Fig. S7(a, b) show the amplitude and phase distribution of the transmission spectrum function with respect to K_x and Ω, respectively. The phase distribution of T exhibits that the vortex in the $k_x - \omega$ domain still survives even with the perturbations, which leads to a zero amplitude at $\omega_0/2\pi = 7.56k$Hz and $k_{0x} = 0.02k_0$. The blue and red circles in Fig. S7(c, d) correspond to the amplitudes and phases of the spectrum function along $K_x/k_0 = 0$ and $\Omega/\omega_0 = 0$ respectively.

S4. Discussion about the calculation of the intrinsic transverse OAM

We note that there is an ongoing theoretical debate about the value of transverse OAM carried by STVPs in Ref. [1, 2, 3]. Depending on the definition of the intrinsic OAM with respect to the photon/phonon probability or energy centroid, this intrinsic OAM (normalized per photon/phonon) equals $\mathrm{L}_\mathrm{y}^\mathrm{int} = l\frac{\gamma+\gamma^{-1}}{2}$ in Ref. [1] and $\mathrm{L}_\mathrm{y}^\mathrm{int} = l\frac{\gamma}{2}$ in Ref. [3], where γ is the ellipticity of the STVP shape in the (z, x) plane. For $\gamma \simeq 7.6$ for the STVP in our experiment, two theoretical values for the intrinsic OAM are about 3.9 and 3.8, respectively. Imperfections of the STVP intensity profile as compared to the ideal elliptical shape make little difference in this case.

Supplementary References

1. Bliokh, K. Y. Orbital angular momentum of optical, acoustic, and quantum-mechanical spatiotemporal vortex pulses. *Phys. Rev. A* **107**, L031501 (2023).
2. Hancock, S. W., Zahedpour, S. & Milchberg, H. M. Mode Structure and Orbital Angular Momentum of Spatiotemporal Optical Vortex Pulses. *Phys. Rev. Lett.* **127**, 193901 (2021).
3. Porras, M. A. Transverse orbital angular momentum of spatiotemporal optical vortices. *arXiv:2301.09105* (2023).

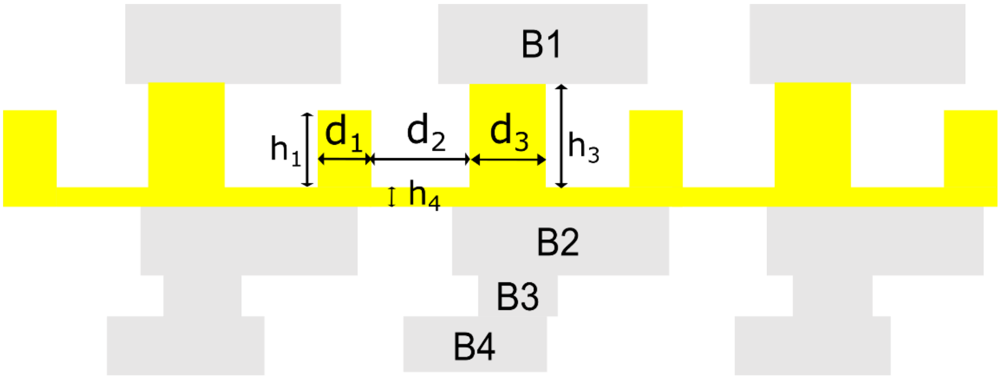

Fig. S1 The schematic of air channels in the meta-grating as shown Fig. 1a in the main text. The block marked in yellow is fixed, and the other four blocks shift toward left or righ

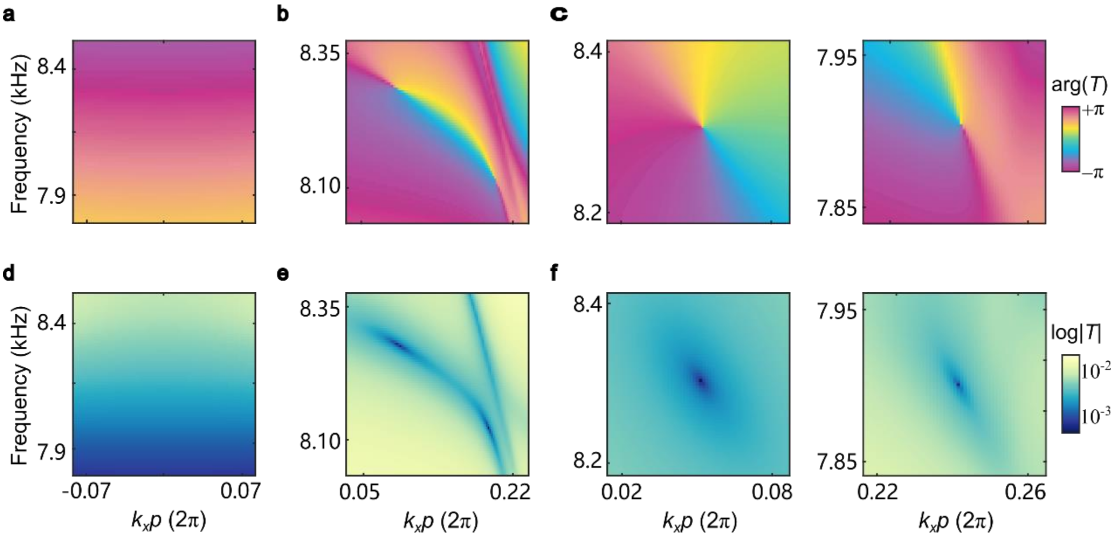

Fig. S2 Simulation data of Fig. 1c-e for the comparison with the experimental results of Fig. 2.

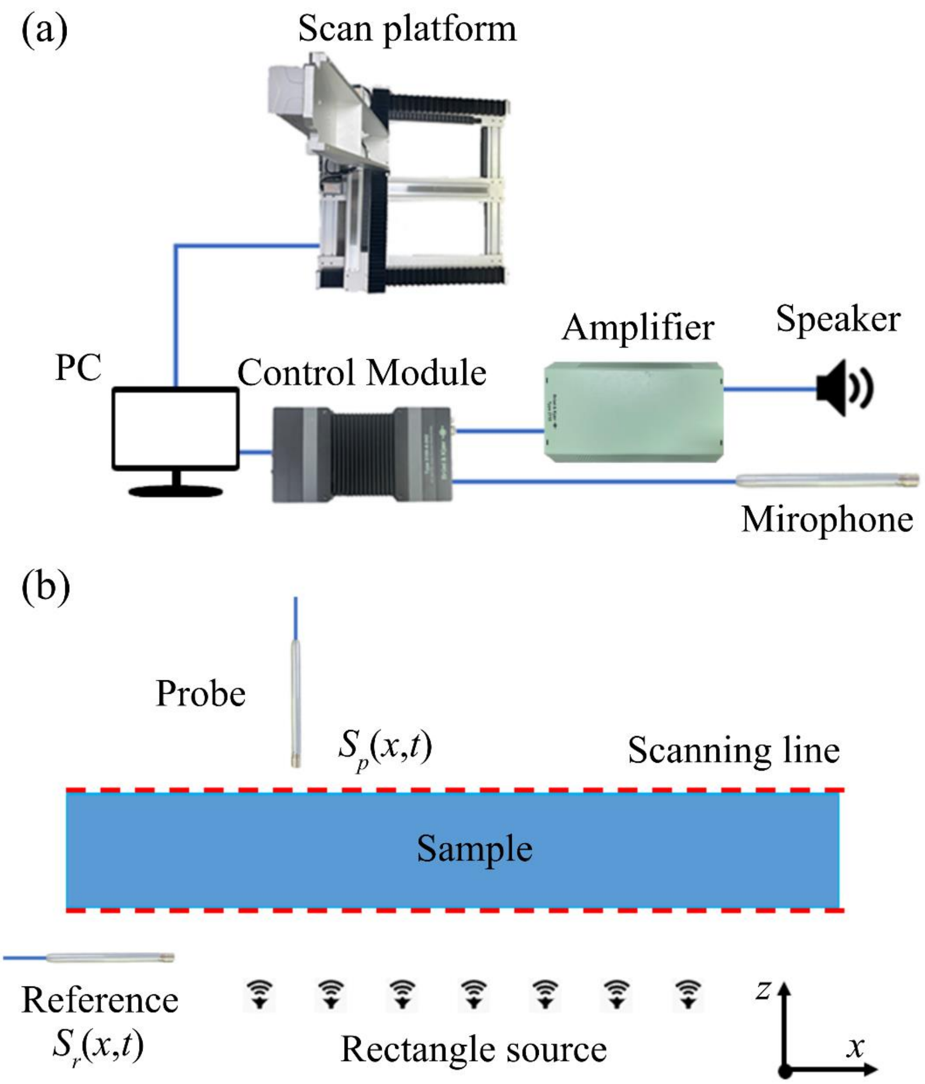

Fig. S3 The experimental setup and the process of measuring. (a) The experimental setup. (b) The process of measuring.

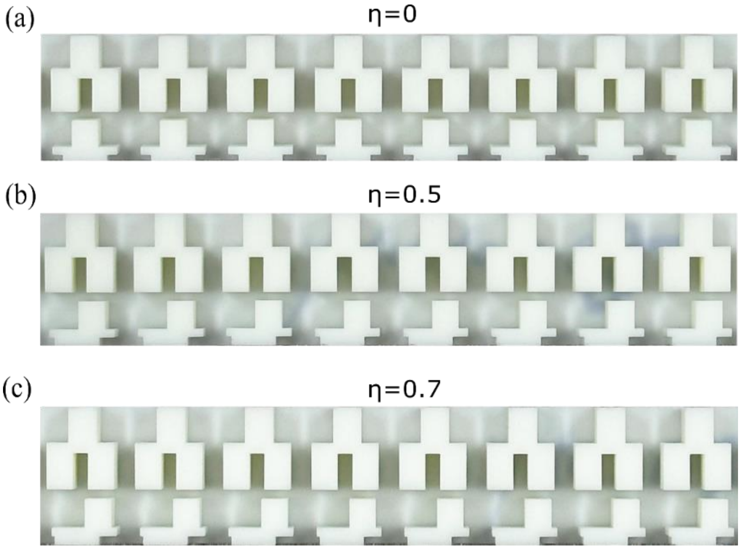

Fig. S4 (a-c) The experimental samples of the meta-gratings for $\eta = 0{,}0.5{,}0.7$.

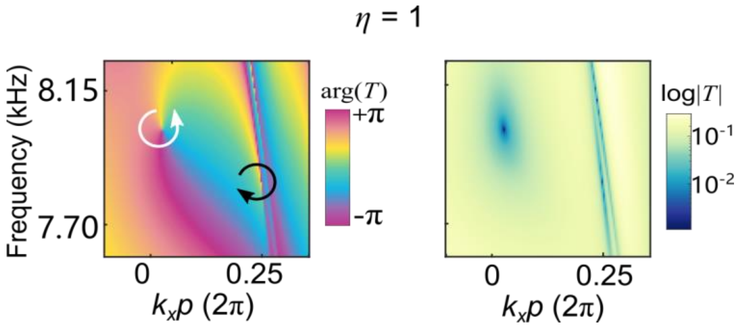

Fig. S5 Numerical simulations for the transmission spectrum function of the meta-grating with $\eta = 1$.

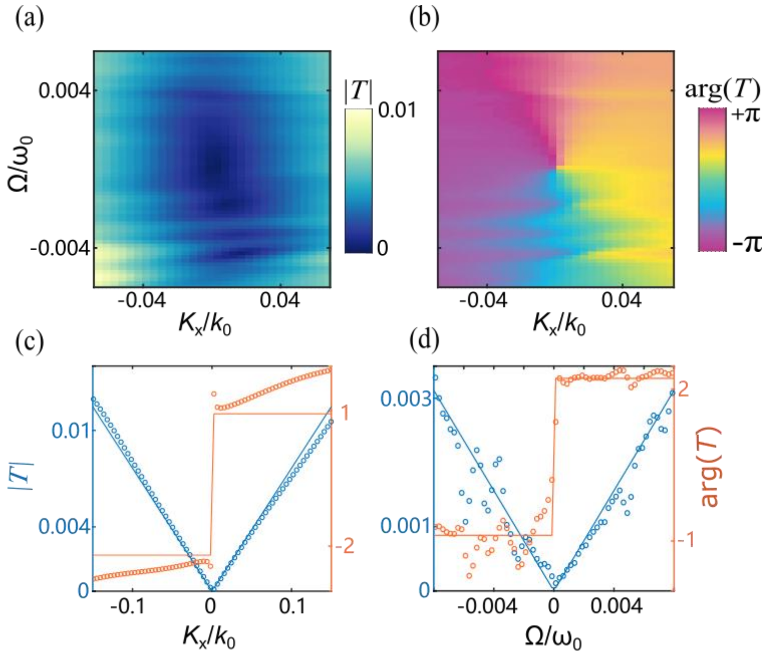

Fig. S6 Measured transmission spectrum function of the unperturbed meta-grating with synthetic parameter $\eta = 1.0$ as Fig. 3b. (a) Amplitude and (b) phase distributions of the spectrum function with respect to K_x and Ω. The amplitudes and phases of the spectrum function along (c) $K_x = 0$ and (d) $\Omega = 0$. The blue circles and lines correspond to the amplitudes of simulation results and the fitting ones with Eq. (S1), and the orange rhombi and lines correspond to the phases, respectively. ω_0 and k_x are the frequency and the wavenumber of light in vacuum at the center wavelength λ_0.

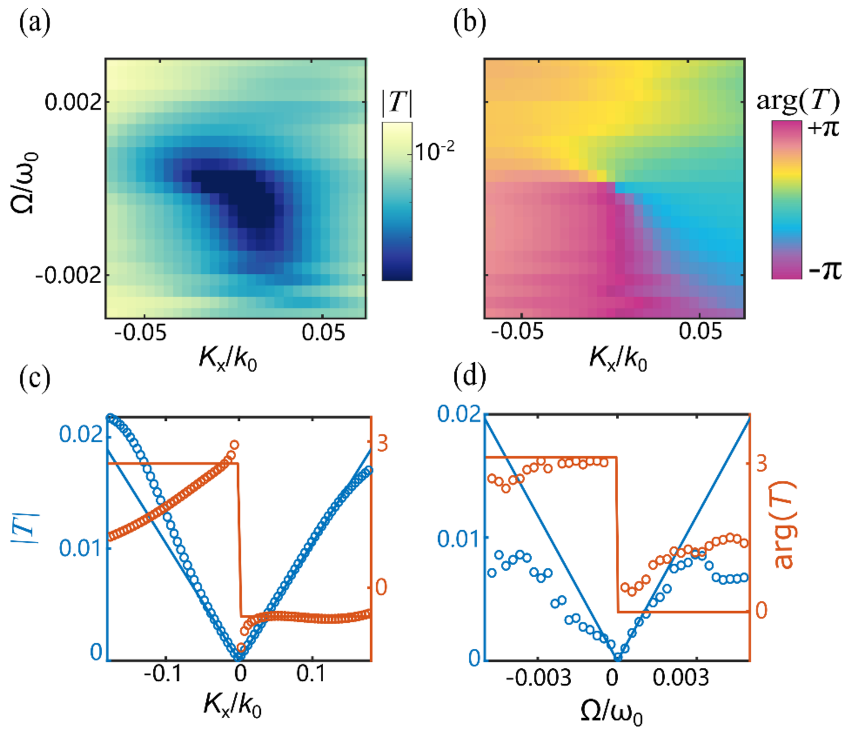

Fig. S7 Transmission spectrum function of the perturbed meta-grating of Fig. 3c, with a block in each unit cell and the randomly placed 16 particles. a) Amplitude and b) phase distributions of the spectrum function with respect to K_x and Ω. The amplitudes and phases of the spectrum function along (c) $K_x = 0$ and (d) $\Omega = 0$. The blue circles and lines correspond to the amplitudes of simulation results and the fitting ones with Eq. (S1), and the orange rhombi and lines correspond to the phases, respectively. ω_0 and k_0 are the frequency and the wavenumber of light in vacuum at the center wavelength λ_0.

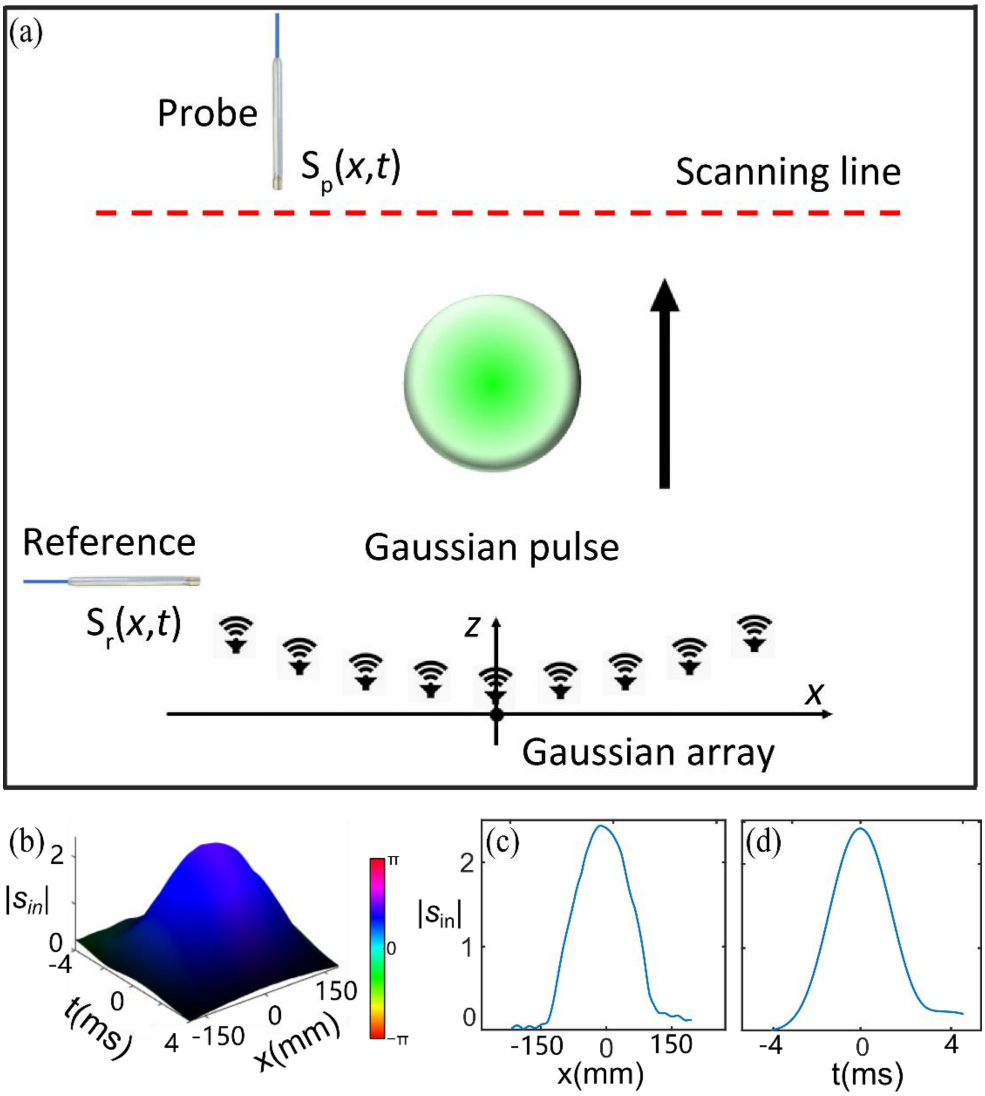

Fig. S8 Experimental setup and the incident pulse which have the Gaussian-like profiles in both the spatial and temporal domains. (a) Experimental setup for measuring STVP. (b) Amplitude and phase distribution of the envelope of the incident pulse. Amplitude distribution of the envelope along (c)$t = 0$ and (d)$x = 0$.

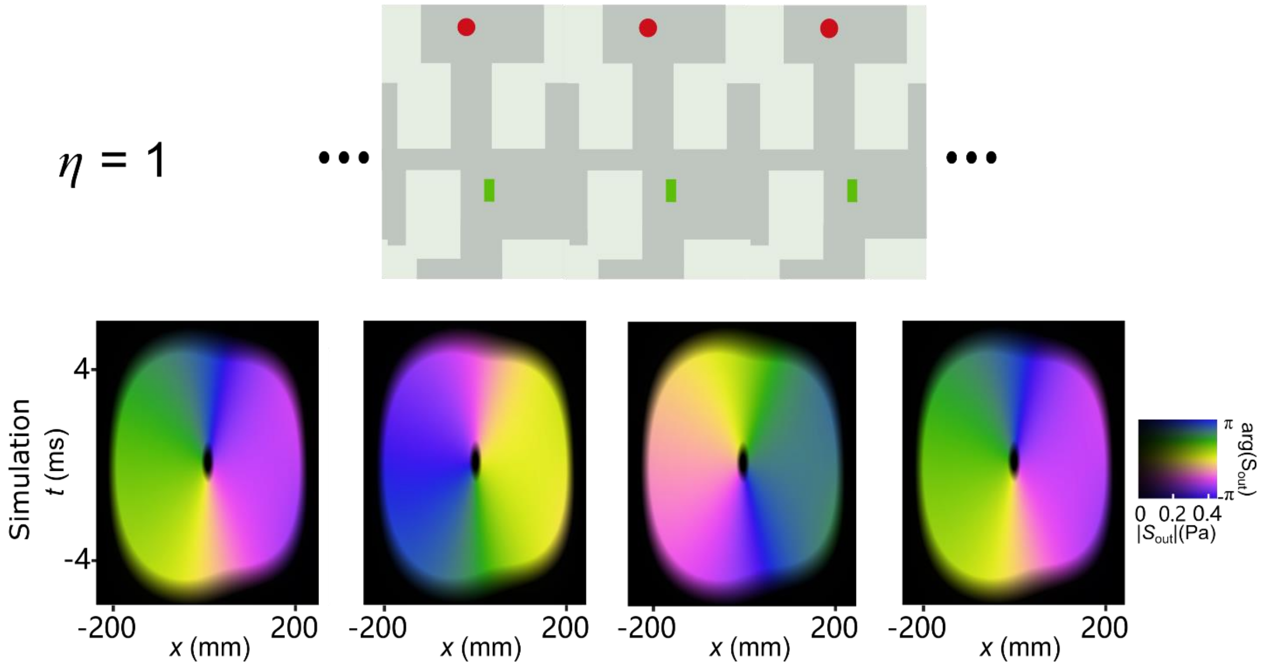

Fig. S9 The simulation results of the evolution of the perturbed spatiotemporal vortex under the perturbated structure with periodic boundary. In the above diagram, the gray area represents the air channel, while the red circles and green rectangles indicate the shape and position of the defects. The below diagram shows the evolution of the perturbed spatiotemporal vortex at the central frequency of the pulse $\omega_0/2\pi$=7.83kHz.
